# High-Strength Bio-Degradable Polymer Foams with Stable High Volume-Expansion Ratio Using Chain Extension and Green Supercritical Mixed-Gas Foaming

**DOI:** 10.3390/polym15040895

**Published:** 2023-02-10

**Authors:** Haoyu Long, Hongsen Xu, Jingwen Shaoyu, Tianchen Jiang, Wei Zhuang, Ming Li, Junyang Jin, Lei Ji, Hanjie Ying, Chenjie Zhu

**Affiliations:** 1 College of Biotechnique and Pharmaceutical Engineering, Nanjing Tech University, No. 30, Puzhu South Road, Nanjing 211816, China; 2 National Engineering Technique Research Center for Biotechnique, Nanjing Tech University, No. 30, Puzhu South Road, Nanjing 211816, China; 3 State Key Laboratory of Materials-Oriented Chemical Engineering, Nanjing Tech University, No. 5, Xinmofan Road, Nanjing 210009, China; 4 Synergetic Innovation Center for Advanced Materials, Nanjing Tech University, No. 30, Puzhu South Road, Nanjing 211816, China

**Keywords:** supercritical foaming, chain extension, poly (butylene adipate-co-terephthalate), polylactic acid, bead foaming

## Abstract

The preparation of biodegradable polymer foams with a stable high volume-expansion ratio (VER) is challenging. For example, poly (butylene adipate-co-terephthalate) (PBAT) foams have a low melt strength and high shrinkage. In this study, polylactic acid (PLA), which has a high VER and crystallinity, was added to PBAT to reduce shrinkage during the supercritical molded-bead foaming process. The epoxy chain extender ADR4368 was used both as a chain extender and a compatibilizer to mitigate the linear chain structure and incompatibility and improve the foamability of PBAT. The branched-chain structure increased the energy-storage modulus (G’) and complex viscosity (η*), which are the key factors for the growth of cells, by 1–2 orders of magnitude. Subsequently, we innovatively used the CO_2_ and N_2_ composite gas method. The foam-shrinkage performance was further inhibited; the final foam had a VER of 23.39 and a stable cell was obtained. Finally, after steam forming, the results showed that the mechanical strength of the PBAT/PLA blended composite foam was considerably improved by the addition of PLA. The compressive strength (50%), bending strength, and fracture load by bending reached 270.23 kPa, 0.36 MPa, and 23.32 N, respectively. This study provides a potential strategy for the development of PBAT-based foam packaging materials with stable cell structure, high VER, and excellent mechanical strength.

## 1. Introduction

As plastic production increases, net emissions from plastic packaging waste incineration will increase to 84 million tons and 309 million tons by 2030 and 2050, respectively [1]. This poses a serious threat to human health and natural ecosystems. Therefore, global efforts are being made to create green polymers from renewable resources. Environmentally friendly plastics provide a possible solution to the above problems [2]. Polymer foam is a polymer/gas composite material with a polymer matrix, which offers the advantages of a lower charge, light weight, high impact strength, and good heat and sound insulation; thus, it is widely used in packaging, construction, medical applications, sound insulation, earthquake resistance, and electromagnetic shielding fields [3,4,5]. Therefore, polymer foam provides an effective mean to alleviate current environmental problems [6]. The foaming industry has focused on research and development of the supercritical bead-foaming technique for poly (butylene adipate-co-terephthalate) (PBAT), polylactic acid (PLA), and other degradable materials, as well as the gradual replacement and elimination of foamed expanded polystyrene and foamed expanded polypropylene [7,8,9].

PBAT is a representative biodegradable material and has been widely used in tableware, plastic bags, agricultural films, and pharmaceutical materials. However, there are few studies and applications of PBAT as a foaming material. Cui et al. prepared biodegradable branched PBAT (CPBAT)/acetylated cellulose nanocrystal (ACNC) nanocomposite foams using a batch foaming process with supercritical CO_2_ [10]. Their study produced the highest volume-expansion ratio (VER) reported for PBAT foam in the literature at that time; however, the VER of their CPBAT foams was only 9.21. PBAT still experiences limiting disadvantages in the field of foaming, such as low melt strength, poor mechanical strength, and low glass-transition temperature (Tg). These shortcomings lead to the low VER of PBAT foams and high shrinkage. Moreover, PBAT foam sheets are soft, which is unsuitable for the field of packaging materials. PLA is a representative biodegradable material with excellent mechanical [11] and physical properties [8,12] and a high foaming VER [13]. PLA supercritical foaming has been extensively studied for a long time. Its development process is mature; however, limitations regarding its molding still exist. Park et al. used PLA beads with a VER of 15 for steam forming; however, the forming interface was poor, which leaves considerable room for improvement in bead molding [14]. The authors proposed that the foaming properties and mechanical strength of PBAT may be improved by mixing PLA into PBAT before foaming [15]. Cai et al. implemented the method of foaming after mixing 75 wt% PBAT and 25 wt% PLA. Under the optimal foaming condition, the VER value of the PBAT/PLA foam was 13.44, and the cell density was 4.08 × 10^8^ cells/cm^3^. Because PBAT and PLA are incompatible systems and both have straight-chain structures and low melt strengths, the VER of unmodified PBAT/PLA is low [16]. Increasing the compatibility between PBAT and PLA is the key to improving the foaming performance of the blend [17,18,19,20]. To the best of our knowledge, there are no existing studies on the preparation of extended chain branched PBAT/PLA composite foams to obtain high-VER foam with PBAT as the main body. Furthermore, the compound-gas foaming and steam-forming properties of PBAT/PLA are also limited.

**Scheme 1:**
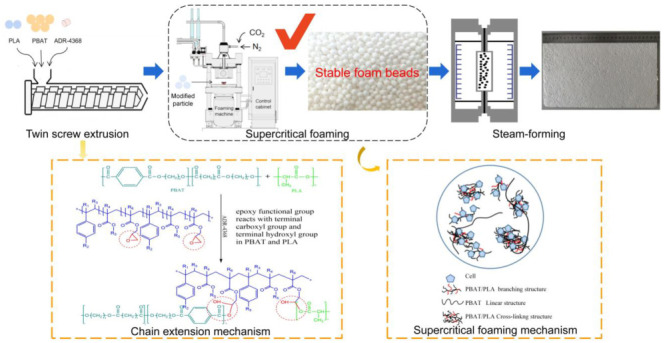
Experiments on supercritical bead composite gas foaming of chain extended PBAT/PLA and reaction-mechanism diagram.

Unmodified PBAT foam has the characteristics of low VER and high shrinkage; it also exhibits poor mechanical properties. In this study, PLA was added to PBAT, and the PBAT/PLA (8:2) branched chain composites were prepared using the twin-screw melt blending method with ADR4368 as a compatibilizer and chain extender (CE). The thermal properties, melt strength, mechanical properties, and crystallization properties of the blend were improved to different degrees, and a comprehensive analysis was conducted. Subsequently, the effects of CO_2_ and N_2_ compound-gas foaming on the shrinkage, cell morphology, and VER evolution of PBAT/PLA (8:2) foam with 1.2% CE was used to adjust the foam performance, and foams with a stable and high VER were obtained at appropriate gas ratios. Finally, a PBAT/PLA composite-foam sheet was prepared using steam-forming technology, and its mechanical strength was considerably improved. This method provides an effective strategy for the study of degradable green foam materials. The foaming process and mechanism used in this study are shown in Scheme 1.

## 2. Experimental Section

### 2.1. Experimental Materials

PBAT (density: 1.24 g/cm^3^, melting point: 95–135 °C) is provided by Shanxi Jinhui Zhaolong High-Tech Co., Ltd., China and its melt index (190 °C/2.16 kg) is 5 g/10 min. PLA (density: 1.24 g/cm^3^, melting point: 155–170 °C) is provided by Foshan Bijia High-Tech Material technique Co., Ltd., China and its melt index (190 °C/2.16 kg) is 1–4 g/10 min. The detailed physical properties and test methods of PBAT and PLA are shown in Table S1. The multifunctional epoxy chain extender ADR4368 is supplied by Badische Anilin-und-Soda-Fabrik (BASF), Germany.

### 2.2. Preparation of Diverse Samples

Branched PBAT/PLA composites were prepared via the twin-screw melt blending method. The diverse samples formulations are shown in Table 1. The details are available in the Section S1.1. For simplicity, for PBAT/PLA samples supplemented with 0 wt%, 0.6 wt%, 0.9 wt%, 1.2 wt%, and 1.5 wt% of the CE, the names are abbreviated as PPCE-0, PPCE-0.6, PPCE-0.9, PPCE-1.2, and PPCE-1.5, respectively.

### 2.3. Preparation of Diverse Types of Foamed Samples

During preparation of diverse types of foamed samples, PPCE-1.2 foam with different gas components was provided for the PPCE-1.2 orthogonal foaming experiment. The diverse samples shown in Table 1 were dried under vacuum at 70 °C for 12 h. The various foams were prepared via an intermittent foaming method in the self-developed supercritical mold foaming equipment using supercritical gas as the physical foaming agent. First, diverse samples were placed inside the high-pressure mold and flushed into the high-pressure mold with saturated gas to 14 MPa and constant foaming temperatures of 105 °C, 110 °C, 115 °C, and 120 °C for 30 min. After waiting for the saturated gas to diffuse and dissolve sufficiently in the matrix, the pressure inside the high-pressure mold dropped from 14 MPa to 0.1 MPa in about 2 s. The orthogonal parallel foaming experiments were performed at different temperatures (110 °C, 115 °C, 120 °C), pressures (12 MPa, 14 MPa, 16 MPa), and saturation times (10 min, 20 min, 30 min) to obtain PPCE-1.2 foams with different foaming conditions.

**Figure 1:**
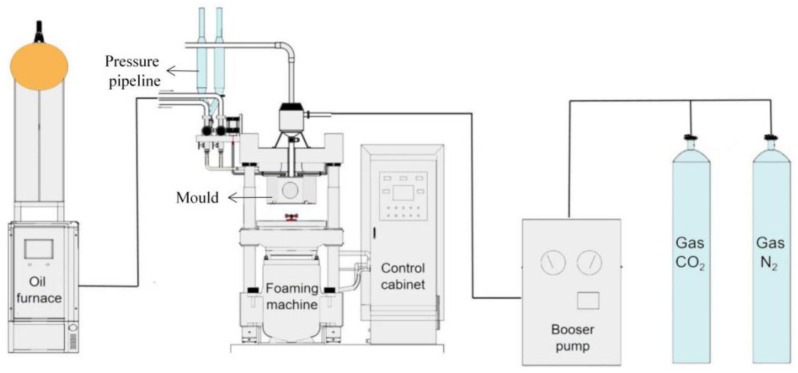
Diagram of supercritical molded bead foaming equipment.

The schematic diagram of the self-developed supercritical mold foaming equipment is shown in Figure 1.

### 2.4. Preparation of Foam Sheets

The PBAT and PPCE-1.2 foam sheets were prepared to use the steam-forming equipment. The PBAT foam sheets and PPCE-1.2 foam sheets obtained under the optimal foaming conditions were sucked into the water steam forming apparatus using a pipeline, the sheets were prepared to use the steam forming equipment, and the molded biodegradable composite sheets were obtained after opening the mold at a double-sided steam pressure of 0.6 kg and 0.9 kg, a heating time of 60 s, a holding time of 10 s, water cooling of 110 s, and a vacuum of 10 s, respectively. The biodegradable composite sheets were obtained after opening the mold.

### 2.5. Material Characterization Analysis

#### 2.5.1. Differential Scanning Calorimetry (DSC) 

DSC (Q20, TA Instruments, New Castle, DE, USA) tested the thermal performance of diverse samples used under a nitrogen flow of 50 mL/min. The details are available in the Section S1.2.1 [21,22]. 

The Crystallinity (*X_c_*) of PBAT was calculated using Equation (1):

(1) $$X_{c\left(PBAT\right)}=\frac{∆H_{m\left(PBAT\right)}}{c_{1}\times ∆H_{m\left(PBAT\right)}^{0}}$$

The Crystallinity (*X_c_*) of *PLA* was calculated using Equation (2):

(2) $$X_{c\left(PLA\right)}=\frac{∆H_{m\left(PLA\right)}-∆H_{cc}}{c_{2}\times ∆H_{m\left(PLA\right)}^{0}}$$

where *X_C(PABT)_* is the degree of crystallinity of PBAT, ∆*H_m(PABT)_* is the PBAT melting enthalpy of various samples, ∆*H_m_*^0^_(*PBAT*)_ is the standard melting enthalpy of the crystallized PBAT (114 J/g) and c_1_ is the mass fraction of PBAT. *X_C(PLA)_* is the degree of crystallinity of PLA, ∆*H_m(PLA)_* is the PLA melting enthalpy of various samples, ∆*H_cc_* is the PLA cold crystallization enthalpy of various samples, ∆*H_m_*^0^*_(PLA)_* is the standard melting enthalpy of the crystallized PLA (93.6 J/g), and *c*_2_ is the mass fraction of PLA.

#### 2.5.2. Thermogravimetric Analysis (TGA) 

The degradation temperatures of diverse samples were tested by a thermogravimetric analyzer (2950, TA Instruments, New Castle, DE, USA). The details are available in the Section S1.2.2.

#### 2.5.3. Avrami Isothermal Crystallization Kinetic

The crystallization kinetic of diverse samples were tested using a DSC (Q20, TA Instruments, New Castle, DE, USA) under a 50 mL/min nitrogen flow rate. The details are available in the Section S1.2.3 [23,24]. 

The Avrami kinetics model was calculated using Equation (3):

(3) $$\ln\left\{-\ln\left[1-X_{\left(t\right)}\right]\right\}=nlnt+lnk$$

The *t*_1/2_ and *G* were calculated using Equations (4) and (5):

(4) $$t_{1/2}={\left(\frac{ln2}{k}\right)}^{1/n}$$

(5) $$G=\frac{1}{t_{1/2}}$$

where *X_(t)_* is the relative crystallinity at crystallization time *t*, *k* is the crystallization kinetic constant for nucleation and growth rate, and *n* is the Avrami index that reflects the crystal nucleation and growth mechanism. 

#### 2.5.4. Rheological Properties

The dynamic rheological performances of diverse samples were tested using a rotational rheometer (MARS40, HAAKE, Germany). The details are available in the Section S1.2.4.

#### 2.5.5. Scanning Electron Microscopy (SEM)

The diverse surface morphologies of non-foamed and foamed samples were obtained using scanning electron microscopy (SEM) (Regulus 8100, Hitachi High-Technologies, Chiyoda Ward, Tokyo, Japan). The details are available in the Section S1.2.5.

#### 2.5.6. Foaming Performance

The volume expansion ratio (*VER*), cell density (*N*_0_), and average cell size (*D*) of diverse foam sample equations and the method for the adsorption capacity of composite gases are provided [25].

The *VER* was calculated using Equation (6):

(6) $$VER=\frac{\rho_{f}}{\rho_{p}}$$

where$\;\rho_{f}$ is the density of unfoamed foam and $\rho_{p}$ is the density of the foamed foam, both in g/cm^3^; the *VER* was measured by an LS12ASCS density balance.

The *N*_0_ was calculated using Equation (7):

(7) $$N_{0}={\left[\frac{nM^{2}}{A}\right]}^{\frac{3}{2}}\times VER$$

where *N*_0_ is the cell density, *n* represents the number of cells in the SEM image, *M* is the magnification of the SEM photograph, A denotes the area (in cm^2^) of the scanning electron microscope (SEM) image, and *VER* denotes the volume expansion ratio.

The *D* was calculated using Equation (8):

(8) $$D=\frac{\sum d_{i}n_{i}}{\sum n_{i}}$$

where *D* is the average cell size, *d_i_* is the individual cell size, and *n_i_* is the number of cell sizes.

#### 2.5.7. Compounding Gas Adsorption

Selection of PPCE-1.2 for gas adsorption and foaming research. The gravimetric method determined the foaming adsorption behaviors at different compound gas ratios. The details are available in the Section S1.2.6.

#### 2.5.8. Mechanical Properties of Sheet Testing

Compressive strength, bending strength, and the fracture load by bending were tested by a universal testing machine (UTM8104, Sanshizongheng Technology Co., Ltd., Shenzhen, China). The details are available in the Section S1.2.7.

## 3. Results and Discussion

### 3.1. Thermal Physical Analysis (DSC)

**Figure 2:**
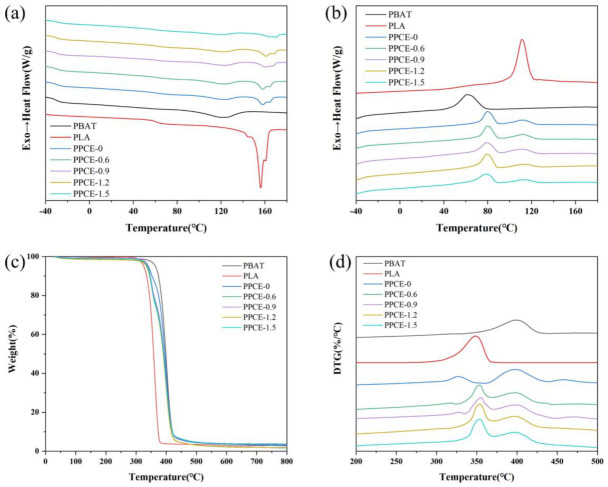
Characterization of different samples. DSC curves of different samples: (**a**) DSC heating curves, (**b**) DSC cooling curves. TGA curves of different samples: (**c**) TG curves, (**d**) DTG curves.

Figure 2a,b show the DSC curves of different samples, and their detailed thermal performance parameters are shown in Table S2. Crystallization and melting properties play a crucial role in the foam cell nucleation, growth, and final foaming properties. This study used DSC analysis at atmospheric pressure to evaluate the crystallization and melting properties of the samples. After blending PBAT/PLA, it was observed that both materials had distinctive DSC peaks, thereby indicating that they could not be mixed into a unified phase. However, the materials influenced each other, and their physical properties changed greatly. As shown in Figure 2a,b and Table S2, the crystallization temperature (*T_c_*) of PBAT was 62.90 °C, and that of PLA was 111.06 °C. The modified PLA with high crystallinity did not exhibit a cold crystallization peak, but had a high *T_c_*, which indicates that it has a high crystallization rate and ensures foam formation. When a 20% proportion of PLA was co-blended in the main body of the PBAT, the *T_c_* corresponding to PBAT was considerably increased (by approximately 17 °C). The *T_c_* of PLA also increased, which may be because the addition of PLA provided both materials with heterogeneous nucleation sites [26], which improved the crystallization rate. Moreover, an interesting phenomenon was observed: the *T_c_* and melting temperature (*T_m_*) of the PBAT phase gradually decreased with the increase in CE content, whereas the *T_c_* and *T_m_* of the PLA phase gradually increased. This may be because the branched reaction reacts more easily with PLA, thereby producing more branched structures and providing crystal nucleation sites. The crystallization behavior of PBAT may be weakened due to the lower chain extension activity of the branched reaction for PBAT. Under the effect of these two mechanisms, the CE exhibits different reactions based on the two phases. This has different effects on the *T_c_*, making the *T_c_* either decrease or increase.

The final crystallinity (*X_c_*) of both the PBAT and PLA phases decreased after their blending and further decreased after the addition of the CE. This is due to the higher chain regularity of the linear structure of PBAT or PLA, which eventually produces a higher *X_c_* under isothermal conditions [27,28]. When complex branched chains are present, the crystal growth is inevitably hindered, due to the increased resistance to molecular motion. This inevitably leads to increased chain entanglement, and the interaction among these crystals weakens the molecular motion in the course of crystal growth, which leads to a lower degree of crystal stacking [29]. The melting-temperature peaks of PBAT and PLA appeared on the heating-scan curves of different PPCE samples, and a double melt peak appeared in the PLA melt peak. This indicates the coexistence of two spherical crystal structures. Additionally, some imperfect crystalline parts melted first and perfect crystalline parts melted later in the process. The *T_g_* of both the PBAT and PLA phases increased after blending PBAT and PLA. Moreover, the *T_g_* values of the PBAT and PLA phases increased with the increase in the CE content after blending. This was due to the entanglement of chain segments, which limited their motion.

### 3.2. Thermogravimetric Analysis (TGA)

Figure 2c,d show the thermal decomposition properties and thermal weight/thermal weight loss (TG/DTG) curves of diverse samples in a nitrogen atmosphere, and detailed thermal decomposition properties are shown in Table S3. In a nitrogen atmosphere, the diverse thermal decomposition of pure PBAT was higher than that of pure PLA, which indicates that the thermal stability of pure PBAT was higher than that of pure PLA. The decomposition peaks of the pure blend, PPCE-0, are the PLA and PBAT decomposition peaks. Moreover, the decomposition temperature of the corresponding thermal decomposition peak of pure PLA decreased by approximately 19 °C, which indicates that the stability of the pure blend PPCE-0 sample decreased considerably due to the incompatibility of the two materials. The thermal weight-loss temperatures of the 5%, 10%, and 50% PPCE samples with the addition of the CE were slightly lower than that of PPCE-0. However, the peak temperature of the thermal decomposition of PLA was approximately 24 °C higher than that without the CE. The thermal decomposition peak temperature of PBAT was essentially unchanged, thereby indicating that the chain extension effect of the CE agent on PLA was pronounced, and the chain extension effect on PBAT was poor. However, the overall thermal stability improved. Moreover, the thermal decomposition performance did not change significantly with an increase in CE content.

### 3.3. Avrami Isothermal Crystallization Kinetic

**Figure 3:**
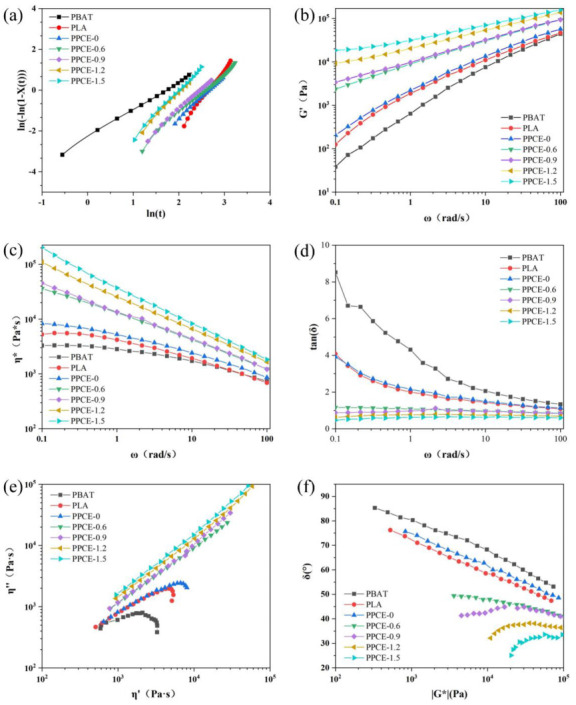
Characterization of samples: (**a**) Kinetics of crystallization of Avrami equation, (**b**) η*, (**c**) G’, (**d**) tan(δ), (**e**) Cole–Cole plots, and (**f**) vGP.

Figure 3a shows the result of the isothermal crystallization analysis of the Avrami model. The detailed parameters of the Avrami model are shown in Table 2. The n values for both PBAT and various PPCE samples were approximately two or lower, thereby indicating that two-dimensional (2D) homogeneous spherical crystal growth dominates the crystallization process. The n values for PLA were approximately three, which indicates that this straight-chain PLA is more inclined to 3D heterogeneous cell nucleation and growth [24,30]. Blending PBAT with PLA alone resulted in a crystallization rate closer to that of the PLA phase, with an overall decrease. As the degree of CE increased, the *t*_1/2_ time shortened. The crystallization rate (G) gradually increased, which is why the two chain-segment groups of PBAT and PLA play the role of crystal heterogeneous nucleation sites, which led to an elevated crystallization rate. However, during this process, the activity of the branched chain reduced, and the crystallization behavior was weakened [31,32]. Overall, the two systems competed and the crystallization rate eventually increased. Therefore, in the overall crystallization process of chain extension PPCE, the chain-segment groups played a dominant role in the effect of crystal heterogeneous nucleation sites, which increased the crystallization rate. Moreover, the crystallization rate of PPCE samples increased with an increase in the chain-expanding agent content.

### 3.4. Rheological Properties

The rheology of polymers is strongly dependent on their melt strength and a vital factor that affects their foaming properties. The shear rheological parameters (G’, η*, and tanδ) of the polymer melt at 190 °C are susceptible to the effects of the polymer molecular chain structure, as well as the type and content of the added filler [33].

Figure 3b shows the storage modulus (G’) of samples with different rheological properties. G’ is a measure of energy storage and recovery that is exhibited during cyclic deformation, and it reflects the elastic moduli of a material [34]. G’ plays a crucial role in polymers’ melt elasticity and foaming properties [35]. The G’ value after the chain extension is more significant than the G’ values for PBAT and PLA, and the G’ values of different samples were elevated over the entire ω range, which is characteristic of the melt elastic behavior of polymers. The branched chains of PBAT and PLA were easily entangled with other PBAT and PLA molecular chains around them after the chain extension. These entanglement points can act as cross-linking points, thereby improving melt strength and foaming properties.

Figure 3c shows the η* values of samples with different rheological properties. The complex viscosity (η*), which reflects the viscosity of the polymer, is closely related to cell nucleation and growth during the foaming process [36], and an increase in η* can assist with preventing cell rupture during the cell-growth stage. Hence, the foaming gas escaped quickly, which resulted in a low foam VER. Different samples exhibited a shear-thinning phenomenon and η* decreased with increasing ω. Compared with pure PBAT and pure PLA, the addition of CE significantly affected the η* value of the PPCE samples. Particularly, at ω = 0.1 rad/s, η* increased substantially by 1–2 orders of magnitude. With an increase in CE content, η* increased and the shear thinning phenomenon became more apparent. This was mainly because the chain extension produced a branched structure, which increased the PPCE sample’s relaxation time and melt strength.

Figure 3d shows the tanδ values of samples with different rheological properties. tanδ is equal to the loss modulus G’’/G’, which has an essential analytical index that reflects the viscoelastic properties of polymers. A larger tanδ value implies a greater viscosity, whereas a smaller tanδ value implies better elasticity. After the addition of the CE, tanδ was significantly lower than that of pure PBAT and pure PLA, thereby indicating a faster elastic response, progressively lower viscous dissipation, and enhanced foamability [37]. This is attributed to the formation of branched structures that increase the number of entanglements between molecular chains.

Cole–Cole and vGP plots were introduced to evaluate the degree of branching and molecular-weight increase in different PPCE samples. Figure 3e shows the Cole–Cole plots of the rheological properties of different samples. A larger radius in the Cole–Cole plot data corresponds to an increased degree of branching [38]. The Cole–Cole plots of PBAT, PLA, and PPCE-0 almost form a semicircle without a tail, thereby confirming their linear structure. The Cole–Cole plot after the chain extension deviates from the trend of the tail rising at higher values of η’ because the value of “η” increases with an increase in η’. This deviation from a semicircle in the Cole–Cole diagram further indicates the appearance of chains with longer relaxation times. This is also the result of physical chain extension and cross-linking, which is a consequence that favors the foaming process.

Figure 3f shows the vGP plots of the rheological properties of different samples. The vGP diagram is typically used when researching the topology of polymers. PBAT, PLA, and PPCE-0 exhibited linear relationships with G *, which confirmed their linear chain structures. The vGP plot of the branched PPCE samples strongly deviated from the trend and exhibited a local extreme in the low-G* range, which implies a nonlinear chain structure [32]. As the CE content increased, the minimum value of the phase angle decreased, which means the molecular weight was more significant. The above results reveal that CE is effective in improving the melt strength.

### 3.5. Phase Morphology of PPCE Samples with Different Degrees of Chain Extension

**Figure 4:**
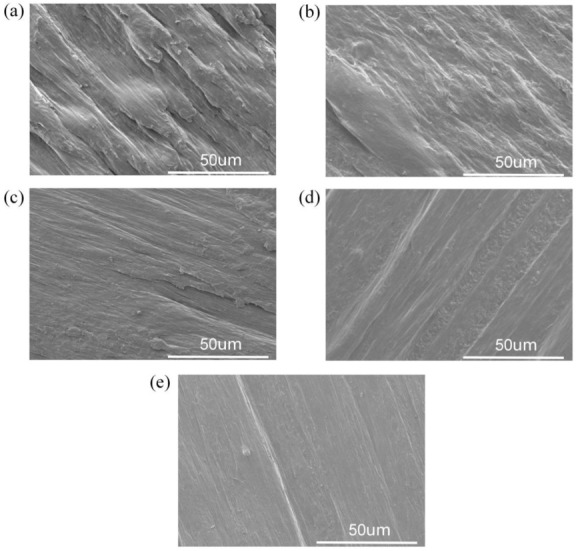
Scanning electron microscopy images of PPCE samples with different degrees of CE: (**a**) PPCE-0, (**b**) PPCE-0.6%, (**c**) PPCE-0.9%, (**d**) PPCE-1.2%, and (**e**) PPCE-1.5%.

Figure 4 shows the scanning electron micrographs of PPCE samples with different degrees of chain extension. Preparing high-performance PBAT/PLA melt composite samples is technically challenging due to the incompatibility of the PBAT and PLA polymers [39]. Figure 4a shows that before the addition of CE, the interfacial adhesion of the PPCE-0 blend was poor, and the microscopic section of the system had many particles. A typical sea island structure should be observed for PPCE-0 blends, which indicates the poor compatibility of PBAT and PLA. The compatibility between PBAT and PLA phases improved with an increase in CE content. The addition of the CE significantly improved the interfacial bonding between PBAT and PLA, even at a lower dosage (0.6 wt%). The compatibility of the PPCE-0.6 interface was also considerably improved. When the CE content reached more than 1.2 wt%, there was no apparent phase separation between PBAT and PLA, the interface between the two phases of the PPCE blend system became blurred, and the number of protruding sheets significantly reduced [40]. The presence of CE may produce PBAT-g-PLA copolymer in the PPCE system, which significantly improves the compatibility of PPCE samples [41].

### 3.6. The VER of Samples under Different Foaming Conditions

All samples were saturated in a 14 MPa CO_2_ atmosphere for 30 min, and the foaming conditions were set at different temperatures; the specific foaming results are shown in Figure S1. Pure PBAT has the lowest VER due to the low melt strength, which limits its foaming effect. The PPCE-0 samples were obtained after the pure blending of PBAT and PLA; the VER improvement was not significant due to the high melting temperature of PLA and incompatibility with PBAT. The foaming performance was substantially improved after different contents of CE were added again. The CE can effectively improve the foaming performance of the PPCE samples; the overall foaming conditions were at their best when the CE content was 1.2%, and the highest VER reached 22.55 at 120 °C. This is because adding CE improves melt strength and the formation of a branched network, which supports stable cell growth and nucleation and effectively improves the compatibility between PBAT and PLA. As the CE content increased to 1.5%, the VER did not continue to increase because a micro-crosslinking phenomenon may occur with the continuous improvement of chain extension. Because the cell wall had a higher strength, the cell growth was more significant than that of PPCE-0.9 and PPCE-1.2. However, the chain activity reduced, cell nucleation weakened, the number of cells significantly reduced, and the supporting effect of foaming weakened; therefore, the VER decreased. Thus, chain extension and blending provide an easy method to prepare high-VER PABT foams for easy handling and large-scale production.

### 3.7. Cell-Morphology Analysis

**Figure 5:**
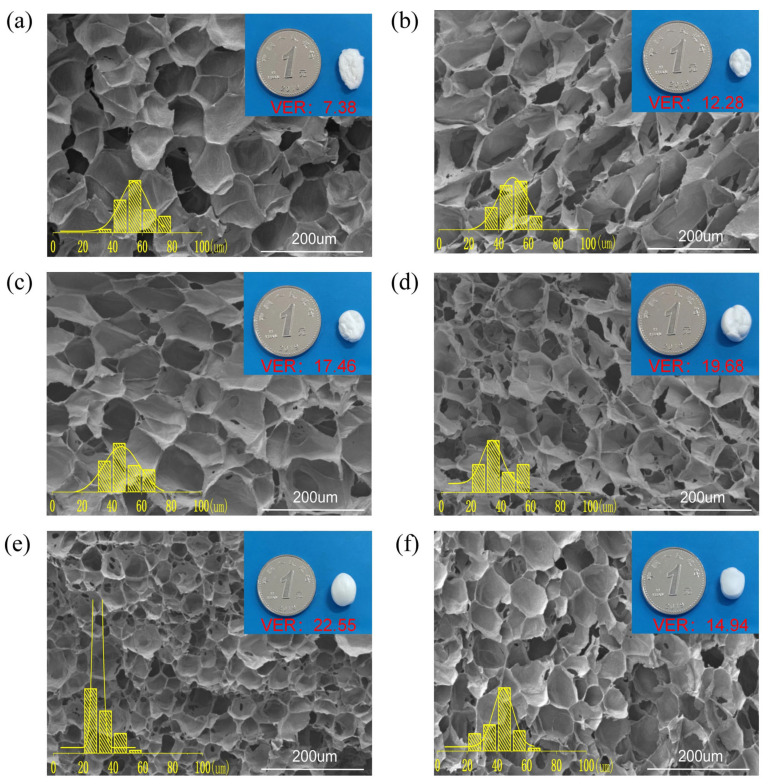
Morphology and size distribution of cells at 120 °C for (**a**) PBAT, (**b**) PPCE-0, (**c**) PPCE-0.6, (**d**) PPCE-0.9, (**e**) PPCE-1.2, and (**f**) PPCE-1.5.

Different degrees of chain extension induce different melt strengths in PPCE samples, affecting cell growth. Figure 5 shows the cell morphology, cell-size distribution, and cell parameters of various foams at a foaming temperature of 120 °C. The detailed parameters are shown in Table 3. The average cell size of PBAT foams was 56.0 μm, the average cell density was 2.62 × 10^7^ cells/cm^3^, and the VER was 7.38. After blending PLA with PBT, the cell size decreased to 49.36 μm, and the cell density increased to 5.27 × 10^7^ cells/cm^3^ because PLA provides heterogeneous nucleation sites that are more favorable for the generation of cells during polymer foaming. Additionally, more complex cell structures emerged after the introduction of the CE [42], and the trend of cell size and cell density is the opposite: the lower the cell density, the larger the cell size [43,44]. Between 0.6% and 1.2% CE, the cell size was smaller, and the cell density increased as the CE content increased. The cell size of PPCE-1.2 reached 31.98 μm with a cell size of 1.85 × 10^8^ cells/cm^3^, which may be due to the improved viscoelasticity of the material. This is due to the branched structure that is formed and the branched chains providing more cell-nucleation sites [45]. When the CE content increased by 1.5%, the cell size and density exhibited different trends from those obtained for CE contents between 0.6% and 1.2%. This may be due to the formation of a more complex cross-linked network at that time; the chain activity was weakened and the nucleation performance was reduced during the foaming process.

### 3.8. Foam-Shrinkage Analysis

Compared to PBAT, PPCE blends exhibited more severe cell rupture due to the incompatibility of PBAT and PLA. Although the CE improved the compatibility of PBAT and PLA, the compatibility was still poor, and the incompatibility at the interfacial bonds could not withstand cell growth. However, the high melt strength provided good support for the growth of the foam. We also observed that PBAT showed severe shrinkage after 110 °C. As the CE content increased, the onset temperature at which the foam began to shrink increased, and foams with PPCE-0, PPCE-0.6, and PPCE-0.9 began to show adhesion and shrinkage at 115 °C. Severe shrinkage occurred at temperatures higher than 120 °C. The material’s melt at this temperature was insufficient to encapsulate the gas, and the cell merge and breakage were detrimental. The PPCE-1.2 and PPCE-1.5 foams showed slight adhesion and shrinkage at 120 °C. The shrinkage of each foam at 120 °C is shown in Figure 5. The addition of PLA inhibited the shrinkage of the material, and the addition of CE further inhibited the shrinkage. The anti-shrinkage property was further strengthened with an increase in CE content. However, foam shrinkage still exists, which limits the further increase in the foam VER. Overcoming this phenomenon is the key to improving the VER.

### 3.9. Foaming Orthogonal and Parallel Experiments

To reduce the foam shrinkage phenomenon, this study investigated the influence of foaming conditions on the foaming state in more detail. We selected PPCE-1.2 samples with the best foaming effect to conduct a more detailed orthogonal foaming study in a reasonable foaming window. The foaming temperature, saturation pressure, and saturation time significantly influence the foam [46]. The orthogonal experiment design of PPCE-1.2 is described in Table S4.

**Figure 6:**
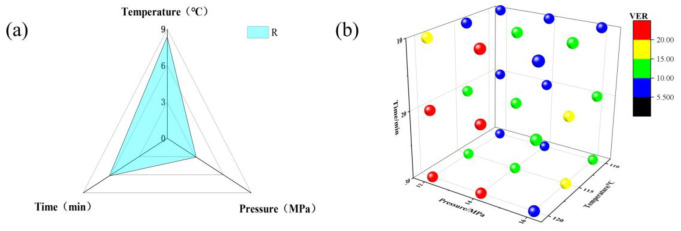
Parallel experiments on PPCE-1.2: (**a**) Orthogonal experiment: R. (**b**) 3D plot of foam VER for three factors.

Figure S2 demonstrates the foaming parallel experiments in a specific interval of foaming conditions; the details are available in Table S5. Figure 6a shows the R-value, and the effect on the foam VER were obtained under the following foaming conditions: temperature > time > pressure. The optimal temperature, pressure, and time foaming conditions were 120 °C, 14 MPa, and 30 min, respectively. Figure 6b shows the 3D plot distribution of the foaming VER for the foaming parallel experiments under three foaming factors; different sphere colors indicate different VER gradients. It is evident from the figure that the foaming window of PPCE-1.2 shown by the yellow and red spheres is the more suitable foaming window.

This parallel experiment verified the foaming orthogonal experiment above, and the highest VER of the foam was 22.55 under the temperature, pressure, and time conditions of 120 °C, 14 MPa, and 30 min, respectively, which is consistent with the results of the orthogonal experiment. Under different foaming conditions, a saturation time of 30 min can complete the saturation process, which is consistent with our previous experimental conclusions. PBAT has an extremely short saturation time compared to that of PLA, which facilitates industrial upscaling and production. We increased the pressure from 14 MPa to 16 MPa; the foam VER decreased sharply, severe shrinkage was observed, and the foam matrix of PPCE-1.2 could no longer continue to support the saturated gas. The foaming state of PPCE-1.2 foam at a temperature, pressure, and time of 120 °C, 14 MPa, and 30 min, respectively, has been described above, and slight adhesion and shrinkage still occur in these conditions.

### 3.10. Compound Gas-Adsorption Capacity

After optimizing the influence of foaming conditions, foam still experiences a shrinkage phenomenon. Zhong et al. obtained TPU sheets with improved mechanical properties and moldability via a steam forming technique using different ratios of CO_2_ and N_2_ compound gas for TPU foaming [47]. The foam-structure stability of TPU foam was improved by compound gas foaming; however, research on PBAT/PLA in compound gas foaming is limited.

To further suppress foam shrinkage, we chose the PPCE-1.2 sample, which exhibited excellent foaming performance. We conducted compound gas foaming experiments under the conditions of a saturation temperature of 120 °C, saturation pressure of 16 MPa, and saturation time of 30 min. The gas adsorption values for various CO_2_/N_2_ ratios are shown in Table S6. The CO_2_/N_2_ ratios described in this study are all pressure ratios. The adsorption of CO_2_ was considerably higher than that of N_2_. CO_2_ had a better affinity with polar polymers and CO_2_ had a higher adsorption amount, which assisted the polymers in forming a homogeneous system [47,48]. However, this also caused a high diffusion rate. Additionally, the T_g_ transition temperature of PBAT is just −28 °C at atmospheric pressure, and the foam was in a highly elastic state at room temperature. The soft texture of PBAT and the fast diffusion of saturated gas were the main reasons for the shrinkage of PBAT foam.

### 3.11. Compounding Gas Foaming-Density Variation

Figure S3 shows the change in density of PPCE-1.2 foam within one week of foaming. For foaming gases of 100%CO_2_, 75%CO_2_ + 25%N_2_, and 50%CO_2_ + 50%N_2_, the initial VERs of all three were similar. The density of the PPCE-1.2 foaming with pure CO_2_ foaming began to increase gradually and reached 0.16 g/cm^3^ after 6 h of maturation after foaming. Then, the density began to decrease and stabilize with the extension of the foaming time. With the increase in N_2_ content in the foaming agent, the density change of PPCE-1.2 became more stable. No significant change in the initial and final densities was observed when the N_2_ content increased by 25% and above at the same pressure, temperature, and saturation time. The foam produced by pure CO_2_ required a long maturation time to ensure the stability of the foam. Additionally, the foams comprising 75% CO_2_ + 25% N_2_ and 50% CO_2_ + 50% N_2_ had smaller densities of 0.053 and 0.057 g/cm^3^, respectively.

### 3.12. Analysis of Compound Gas-Foaming Morphology and Cells

**Figure 7:**
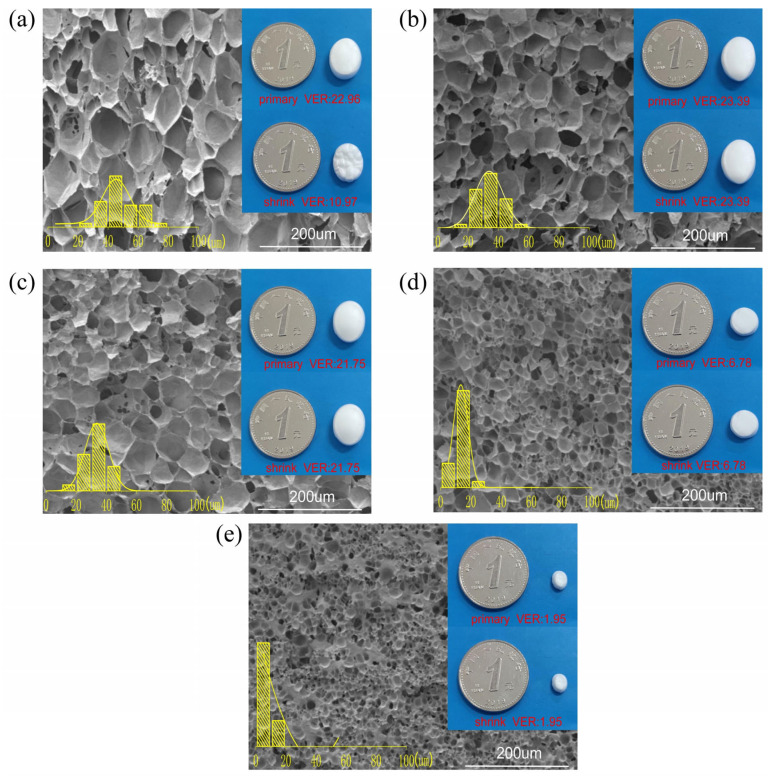
Distribution of cell morphology and cell size of PPCE-1.2 samples under different gas conditions (**a**) 100% CO_2_, (**b**) 75% CO_2_ + 25% N_2_, (**c**) 50% CO_2_ + 50% N_2_, (**d**) 25% CO_2_ + 75% N_2_, and (**e**) 100% N_2_.

Figure 7 demonstrates the cell morphology, size distribution, and parameters of PPCE-1.2 samples under different gas conditions, and detailed cell parameters are listed in Table 4. Compared with the previous foaming conditions, we increased the pressure to 16 MPa, and a severe shrinkage phenomenon occurred. This indicates that the critical value of the cell tolerable gas had been reached. Therefore, we used compound gas foaming to suppress the shrinkage of the foams. Under this foaming condition, the VER of a pure CO_2_ foamed PPCE-1.2 sample was reduced from 22.96 to 10.97; the sample exhibited 52.22% shrinkage, which is considered to be severe shrinkage, and surface wrinkling. For the 75% CO_2_ + 25% N_2_ compound gas foaming, the cell size, cell density, and VER of the foam were 34.10 μm, 1.78 × 10^8^ cells/cm^3^, and 23.39, respectively. When 50% N_2_ was added, the cell size, cell density, and VER of the 50% CO_2_ + 50% N_2_ compound gas foaming were similar to those of the 75% CO_2_ + 25% N_2_ compound gas foaming. As the proportion of N_2_ continued to increase, the VER decreased sharply to just 6.78, and the cell density of the 25% CO_2_ + 75% N_2_ compound gas foaming improved by an order of magnitude compared to that of the pure CO_2_ foaming. As the N_2_ content increased from 0% to 75%, the cell size continued to decrease and the cell density increased. This is because N_2_ has a more vital nucleation ability than CO_2_, which promotes the generation of nucleation sites in the foaming process [49]. When pure N_2_ is used as a foaming agent, the cell density exhibits a decreasing tendency. The cell structure is complex, and the cell wall is thick, which is relevant to the apparent unfoamed area between the cells. Additionally, the foam VER is low, only 1.95, because the driving force of N_2_ on cell growth is lower than that of CO_2_. However, a saturation time of 30 min is insufficient when pure N_2_ is used as the foaming gas.

Supercritical CO_2_ foaming produces a wider distribution of cell sizes and a higher proportion of large and small cells. This is due to the superior absorption of CO_2_, which facilitates the growth of cells. The driving force of N_2_ on cell growth is smaller, and the adsorption amount is smaller. Cell nucleation is increased, which results in a larger number of cells at a smaller size. Additionally, the cell size distribution is in a general range [19,49]. Overall, CO_2_ and N_2_ compound foaming can effectively inhibit the shrinkage of cells. The compound gas foaming of 75% CO_2_ + 25% N_2_ is the most effective.

### 3.13. Sheet Forming and Mechanical Property Analysis

**Figure 8:**
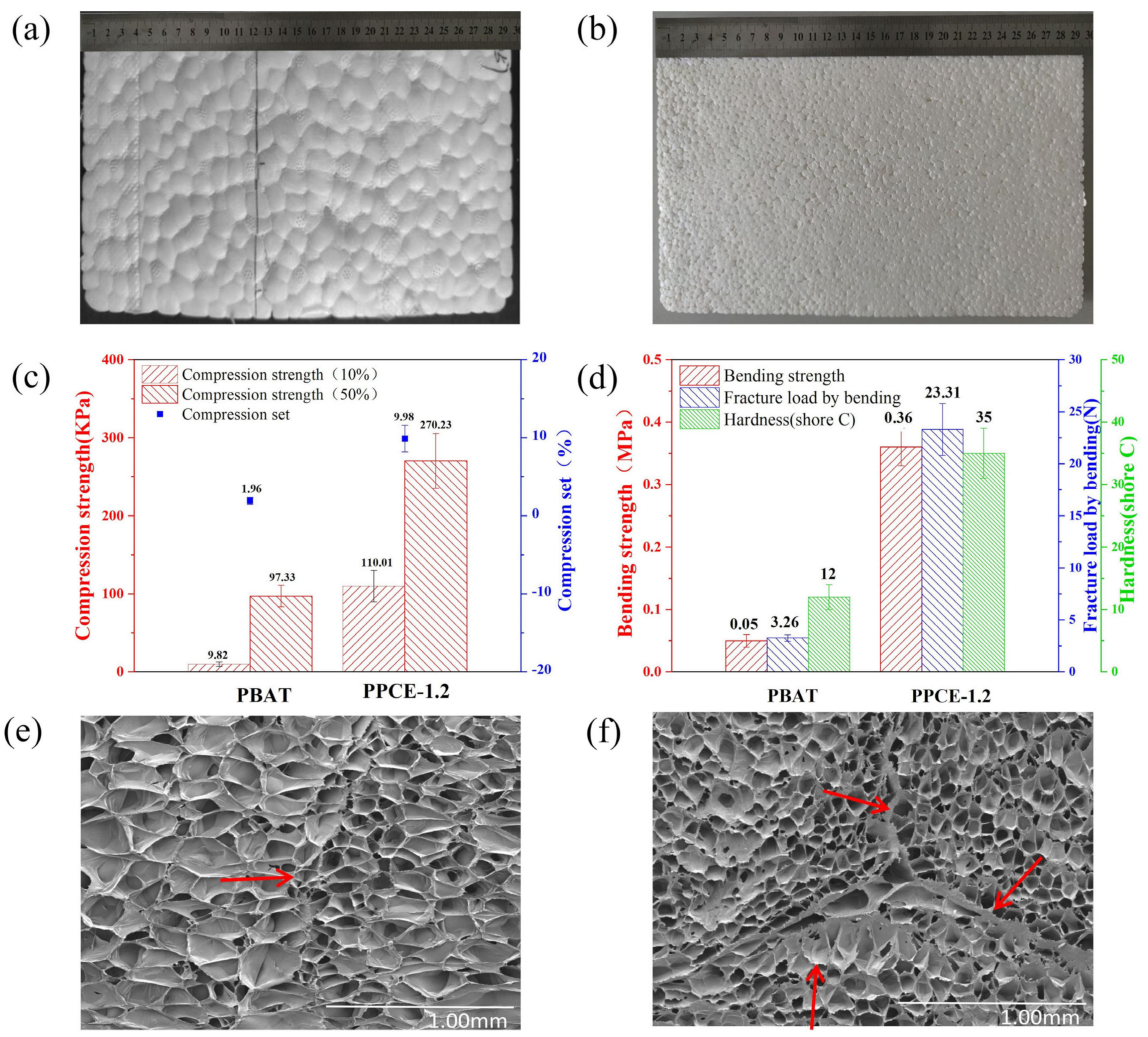
(**a**) PBAT foam-forming sheets and (**b**) PPCE-1.2 foam-forming sheets. Characterization of PBAT and PPCE-1.2: (**c**) compression properties, (**d**) bending properties and hardness (shore C). (**e**) SEM image of PBAT foaming particle-bonding position. (**f**) SEM image of PPCE-1.2 foaming particle-bonding position.

Figure 8a,b show the PBAT and PPCE-1.2 foam-forming sheets, respectively. Steam-forming uses high temperatures at a particular pressure to melt the foam and adhere it together while eliminating the voids in the foam. The key contribution of steam forming is that the polymer chains diffuse to the interface area of the foam bead and rearrange under high-temperature steam [20,49]. The densities of the PBAT and PPCE-1.2 foam sheets obtained using steam-forming equipment under the optimal foaming conditions were 0.128 g/cm^3^ and 0.113 g/cm^3^, respectively. The final molding densities of the two were relatively close.

Figure 8c shows the compression strength and permanent compression deformation of the PBAT and PPCE-1.2 foam sheets. PBAT has low Tg, excellent toughness, and a high elastic state. Therefore, its molded sheets were incredibly soft and had insufficient compressive strength at 10% and 50% deformation, which led to minor permanent compression deformation. The mechanical and physical properties of PLA were excellent. After adding 20% PLA and 1.2% CE to PBAT, the compressive strength of the foam significantly increased. Moreover, the high hardness, low resilience, and high brittleness of PLA caused the permanent compression deformation of PPCE-1.2 to increase, but kept the permanent deformation low [20,50].

Figure 8d shows the bending strength, the fracture load demonstrated by bending, and the shore hardness C of the PBAT and PPCE-1.2 foam sheets. As previously mentioned, adding PLA increased the shore hardness C of the foam board from 12 to 35. Notably, the PBAT foam sheet was exceptionally soft and exhibited high toughness. No fracturing occurred during bending, due to the low bending strength and fracture load demonstrated by bending. The low mechanical strength limited the foam’s application in packaging and other fields. After the PPCE-1.2 foam sheet was prepared, the bending strength increased from 0.05 M Pa to 0.36 M Pa, and the fracture load by bending increased from 3.26 MPa to 23.31 MPa, which effectively expanded the application range of the foaming sheet.

Figure 8e,f show the SEM images of PBAT and PPCE-1.2 foam bonding, respectively. The interface thickness of PPCE-1.2 was thicker than that of PBAT in terms of the foam-forming joint, and the interface part that did not fuse well after molding provided more fracture points in the mechanical property testing. Therefore, adding PLA led to a higher mechanical strength in the foam plate. Moreover, the cell density of PPCE-1.2 was higher and the cell size was smaller, which plays a more important role in supporting the foam and preventing the bursting and shrinkage of the foam. Therefore, a plate with excellent mechanical properties was produced [51].

## 4. Conclusions

In conclusion, a simple, clean, and highly efficient preparation method of PBAT/PLA degradable green foam material is presented. It has the following advantages. (1) It used ADR4368 as a CE and compatibilizer to improve the foaming properties, compatibility, rheological properties, and thermal properties of PPCE composite samples; compared with the system without chain extender, the VER increased from 7.38 to 22.55. (2) After using composite gas to coordinate the process of cell nucleation and cell growth, the cell size reduced, the cell density increased, no foams shrinkage was observed, and the VER further increased to 23.39. (3) For the foam sheets obtained via the steam-forming technique, compared with pure PBAT sheets, the compressive strength (50%) increased from 97.33 kPa to 270.23 kPa, the bending strength increased from 0.05 MPa to 0.36 MPa, and the fracture load obtained via bending increased from 12 N to 23.32 N. Hence, we believe that this strategy will find wide applications in the green packaging field, as well as in industry.

## Figures and Tables

**Table 1:** Experimental formulae of different PBAT, PLA, and PBAT/PLA blend samples.

Sample Name	PBAT (wt%)	PLA (wt%)	CE (wt%)
PBAT	100	0	0
PLA	0	100	0
PPCE-0	80	20	0
PPCE-0.6	80	20	0.6
PPCE-0.9	80	20	0.9
PPCE-1.2	80	20	1.2
PPCE-1.5	80	20	1.5

**Table 2:** Semi-crystallization time, crystallization rate, and Avrami-model parameters for different samples at atmospheric pressure and a temperature of 90 °C.

Sample	t_1/2_/min ^a^	G/min^−1 b^	n ^c^	lnk ^d^	k ^e^
PBAT	4.2	0.238	1.34	−2.29	1.01 × 10^−1^
PLA	12.52	0.08	2.85	−7.57	5.16 × 10^−4^
PPCE-0	12.03	0.083	2.06	−5.49	4.13 × 10^−3^
PPCE-0.6	10.89	0.092	1.89	−4.88	7.60 × 10^−3^
PPCE-0.9	9.74	0.103	2.04	−5.01	6.67 × 10^−3^
PPCE-1.2	6.65	0.15	2.25	−4.63	9.75 × 10^−3^
PPCE-1.5	6.3	0.159	2.24	−4.49	1.12 × 10^−2^

^a^ Half crystallization time; ^b^ Crystallization rate; ^c^ Avrami index; ^d^ Log of Kinetic constant; ^e^ Kinetic constant.

**Table 3:** Foam parameters of PPCE samples with different degrees of chain extension at a foaming temperature of 120 °C.

Sample	PBAT	PPCE-0	PPCE-0.6	PPCE-0.9	PPCE-1.2	PPCE-1.5
Cell size (μm)	56.01	49.36	49.92	39.13	31.98	42.34
Cell density (10^7^ cells/cm^3^)	2.62	5.27	7.36	11.96	18.5	8.07
Density (g/cm^3^)	0.168	0.101	0.071	0.063	0.055	0.083
VER	7.38	12.28	17.46	19.68	22.55	14.94

**Table 4:** Cell parameters of PPCE-1.2 samples under different gas conditions.

Sample	100%CO_2_	75%CO_2_ + 25%N_2_	50%CO_2_ + 50%CO_2_	25%CO_2_ + 75%N_2_	100%N_2_
Cell size (μm)	47.79	34.10	33.06	12.00	8.29
Cell density (10^7^ cells/cm^3^)	4.87	17.75	18.69	24.18	12.16
Density (g/cm^3^)	0.113	0.053	0.057	0.183	0.636
VER	10.97	23.39	21.75	6.78	1.95

## Data Availability

Not applicable.

## Supplementary Materials

The following supporting information can be downloaded at: https://www.mdpi.com/article/10.3390/polym15040895/s1, Table S1. PBAT and PLA detailed property tables and test standard; Table S2. Thermal performance parameter of PBAT, PLA, and diverse PPCE samples; Table S3. Thermal decomposition properties of diverse samples; Table S4. Orthogonal experiment design of PPCE-1.2; Table S5. Results of foaming parallel experiments in a specific interval of foaming conditions; Table S6. CO_2_ and N_2_ gas adsorption amounts; Figure S1. Diverse samples saturated with 14 MPa CO_2_ saturated atmosphere for 30 min and foamed at different foaming temperatures; Figure S2. Parallel experiments on PPCE-1.2: (a) 110 °C, (b) 115 °C, (c) 120 °C; Figure S3. Density variation of foam maturation process under different gas conditions.

## References

[1] Shen M., Huang W., Chen M., Song B., Zeng G., Zhang Y. (2020). (Micro)plastic crisis: Un-ignorable contribution to global greenhouse gas emissions and climate change. J. Clean. Prod..

[2] Peng K., Mubarak S., Diao X., Cai Z., Zhang C., Wang J. (2022). Progress in the preparation, properties, and applications of PLA and its composite microporous materials by supercritical CO_2_: A review from 2020 to 2022. Polymers.

[3] Fei Y., Chen F., Fang W., Xu L., Ruan S., Liu X., Zhong M., Kuang T. (2020). High-strength, flexible and cycling-stable piezo-resistive polymeric foams derived from thermoplastic polyurethane and multi-wall carbon nanotubes. Compos. Part B Eng..

[4] Liu J., Zhang L., Shun W., Dai J., Peng Y., Liu X. (2021). Recent development on bio-based thermosetting resins. J. Polym. Sci..

[5] Aversa C., Barletta M., Cappiello G., Gisario A. (2022). Compatibilization strategies and analysis of morphological features of poly(butylene adipate-co-terephthalate) (PBAT)/poly(lactic acid) PLA blends: A state-of-art review. Eur. Polym. J..

[6] Faba S., Arrieta M.P., Aguero A., Torres A., Romero J., Rojas A., Galotto M. (2022). Processing compostable PLA/organoclay bionanocomposite foams by supercritical CO_2_ foaming for sustainable food packaging. Polymers.

[7] Ameli A., Nofar M., Jahani D., Rizvi G., Park C.B. (2015). Development of high void fraction polylactide composite foams using injection molding: Crystallization and foaming behaviors. Chem. Eng. J..

[8] Nofar M., Ameli A., Park C.B. (2015). A novel technology to manufacture biodegradable polylactide bead foam products. Mater. Des..

[9] Liao J., Zhang S., Wang Z., Song X., Zhang D., Kumar R., Jin J., Ren P., You H., Chen F. (2020). Transition-metal catalyzed asymmetric reactions under continuous flow from 2015 to early 2020. Green Synth. Catal..

[10] Cui Y., Luo J., Deng Y., Wang X., Zhou H. (2021). Effect of acetylated cellulose nanocrystals on solid-state foaming behaviors of chain-extended poly(butylene adipate-co-terephthalate). J. Vinyl Addit. Technol..

[11] Jiang L., Wolcott M.P., Zhang J. (2006). Study of Biodegradable/Poly(butylene adipate-co-terephthalate) blends. Biomacromolecules.

[12] Nofar M., Park C.B. (2014). Poly (lactic acid) foaming. Prog. Polym. Sci..

[13] Li Y., Yin D., Liu W., Zhou H., Zhang Y., Wang X. (2020). Fabrication of biodegradable poly (lactic acid)/carbon nanotube nanocomposite foams: Significant improvement on rheological property and foamability. Int. J. Biol. Macromol..

[14] Nofar M., Tabatabaei A., Sojoudiasli H., Park C.B., Carreau P.J., Heuzey M.C., Kamal M.R. (2017). Mechanical and bead foaming behavior of PLA-PBAT and PLA-PBSA blends with different morphologies. Eur. Polym. J..

[15] Li Y., Zhang Z., Wang W., Gong P., Yang Q., Park C.B., Li G. (2022). Ultra-fast degradable PBAT/PBS foams of high performance in compression and thermal insulation made from environment-friendly supercritical foaming. J. Supercrit. Fluids.

[16] Cai W., Liu P., Bai S., Li S. (2021). A one-step method to manufacture biodegradable poly (butylene adipate-co-terephthalate) bead foam parts. Polym. Adv. Technol..

[17] Nofar M., Salehiyan R., Ciftci U., Jalali A., Durmuş A. (2020). Ductility improvements of PLA-based binary and ternary blends with controlled morphology using PBAT, PBSA, and nanoclay. Compos. Part B Eng..

[18] Wang X., Peng S., Chen H., Yu X., Zhao X. (2019). Mechanical properties, rheological behaviors, and phase morphologies of high-toughness PLA/PBAT blends by in-situ reactive compatibilization. Compos. Part B Eng..

[19] Chen Y., Li D., Zhang H., Ling Y., Wu K., Liu T., Hu D., Zhao L. (2021). Antishrinking strategy of microcellular thermoplastic polyurethane by comprehensive modeling analysis. Ind. Eng. Chem. Res..

[20] Ge C., Ren Q., Wang S., Zheng W., Zhai W., Park C.B. (2017). Steam-chest molding of expanded thermoplastic polyurethane bead foams and their mechanical properties. Chem. Eng. Sci..

[21] Bastarrachea L., Dhawan S., Sablani S.S., Mah J.H., Kang D.H., Zhang J., Tang J. (2010). Biodegradable poly(butylene adipate-co-terephthalate) films incorporated with nisin: Characterization and effectiveness against listeria innocua. J. Food Sci..

[22] Fischer E.W., Sterzel H.J., Wegner G. (1973). Investigation of the structure of solution grown crystals of lactide copolymers by means of chemical reactions. Colloid Polym. Sci..

[23] Colton J.S., Suh N.P. (1987). The nucleation of microcellular thermoplastic foam with additives: Part 1: Theoretical considerations. Polym. Eng. Sci..

[24] Cai J., Liu M., Wang L., Yao K., Li S., Xiong H. (2011). Isothermal crystallization kinetics of thermoplastic starch/poly(lactic acid) composites. Carbohydr. Polym..

[25] Avrami M. (1940). Kinetics of phase change. II transformation-time relations for random distribution of nuclei. J. Chem. Phys..

[26] Zhang R., Xiong Y., Liu Q., Hu S. (2017). Improved cell morphology and thermal properties of expanded polypropylene beads by the addition of PP with a high melting point. J. Appl. Polym. Sci..

[27] Tian J., Yu W., Zhou C. (2011). Crystallization kinetics of linear and long-chain branched polypropylene. J. Macromol. Sci. Part B: Phys..

[28] Pilla S., Kim S.G., Auer G.K., Gong S., Park C.B. (2009). Microcellular extrusion-foaming of polylactide with chain-extender. Polym. Eng. Sci..

[29] Ouchi T., Ichimura S., Ohya Y. (2006). Synthesis of branched poly (lactide) using polyglycidol and thermal, mechanical properties of its solution-cast film. Polymer.

[30] Nofar M., Zhu W., Park C.B. (2012). Effect of dissolved CO_2_ on the crystallization behavior of linear and branched PLA. Polymers.

[31] Zhang C., Lan Q., Zhai T., Nie S., Luo J., Yan W. (2018). Melt crystallization behavior and crystalline morphology of polylactide/poly(epsilon-caprolactone) blends compatibilized by lactide-caprolactone copolymer. Polymers.

[32] Qiao Y., Li Q., Jalali A., Yang J., Wang X., Zhao N., Jiang Y., Wang S., Hou J., Jiang J. (2021). In-situ microfibrillated poly(ε-caprolactone)/poly(lactic acid) composites with enhanced rheological properties, crystallization kinetics and foaming ability. Compos. Part B Eng..

[33] Yin D., Mi J., Zhou H., Wang X., Tian H. (2020). Fabrication of branching poly (butylene succinate)/cellulose nanocrystal foams with exceptional thermal insulation. Carbohydr. Polym..

[34] Pradeep S.A., Kharbas H., Turng L.S., Avalos A., Lawrence J.G., Pilla S. (2017). Investigation of thermal and thermomechanical properties of biodegradable PLA/PBSA composites processed via supercritical fluid-assisted foam injection molding. Polymers.

[35] Shi X., Qin J., Wang L., Ren L., Rong F., Li D., Wang R., Zhang G. (2018). Introduction of stereocomplex crystallites of PLA for the solid and microcellular poly(lactide)/poly(butylene adipate-co-terephthalate) blends. RSC Adv..

[36] Chen Z., Hu J., Ju J., Kuang T. (2019). Fabrication of poly(butylene succinate)/carbon black nanocomposite foams with good electrical conductivity and high strength by a supercritical CO_2_ foaming process. Polymers.

[37] He H., Wang G., Chen M., Xiong C., Li Y., Tong Y. (2020). Effect of different compatibilizers on the properties of poly (lactic acid)/poly (butylene adipate-co-terephthalate) blends prepared under intense shear flow field. Materials.

[38] Tian H., Wang Z., Jia S., Pan H., Han L., Bian J., Li Y., Yang H., Zhang H. (2021). Biodegradable foaming material of poly(butylene adipate-co-terephthalate) (PBAT)/poly(propylene carbonate) (PPC). Chin. J. Polym. Sci..

[39] Sun M., Zhang L., Li C. (2022). Modified cellulose nanocrystals based on SI-ATRP for enhancing interfacial compatibility and mechanical performance of biodegradable PLA/PBAT blend. J. Polym. Compos..

[40] Zhou W., Rao Y., Zhuang W., Ge L., Lin R., Tang T., Wu J., Li M., Yang P., Zhu C. (2021). Improved enzymatic activity by oriented immobilization on graphene oxide with tunable surface heterogeneity. Compos. Part B Eng..

[41] Wu D., Huang A., Fan J., Xu R., Liu P., Li G., Yang S. (2021). Effect of blending procedures and reactive compatibilizers on the properties of biodegradable poly(butylene adipate-co-terephthalate)/poly(lactic acid) blends. J. Polym. Eng..

[42] Cui Y., Zhou H., Yin D., Zhou H., Wang X. (2020). An innovative strategy to regulate bimodal cellular structure in chain extended poly(butylene adipate- co- terephthalate) foams. J. Vinyl Addit. Technol..

[43] Kuang T., Li K., Chen B., Peng X. (2017). Poly (propylene carbonate)-based in situ nanofibrillar biocomposites with enhanced miscibility, dynamic mechanical properties, rheological behavior and extrusion foaming ability. Compos. Part B Eng..

[44] Gao C., Li M., Zhu C., Hu Y., Shen T., Li M., Ji X., Lyu G., Zhuang W. (2021). One-pot depolymerization, demethylation and phenolation of lignin catalyzed by HBr under microwave irradiation for phenolic foam preparation. Compos. Part B Eng..

[45] Wang X., Mi J., Zhou H., Wang X. (2018). Transition from microcellular to nanocellular chain extended poly(lactic acid)/hydroxyl-functionalized graphene foams by supercritical CO_2_. J. Mater. Sci.

[46] Zhou C., Wang P., Li W. (2011). Fabrication of functionally graded porous polymer via supercritical CO_2_ foaming. Compos. Part B Eng..

[47] Zhong W., Yu Z., Zhu T., Zhao Y., Phule A.D., Zhang Z. (2022). Influence of different ratio of CO_2_/N_2_ and foaming additives on supercritical foaming of expanded thermoplastic polyurethane. Express Polym Lett..

[48] Horne W.J., Shannon M.S., Bara J.E. (2014). Correlating fractional free volume to CO_2_ selectivity in [Rmim][Tf_2_N] ionic liquids. J. Chem. Thermodyn..

[49] Li R., Lee J., Wang C., Mark L.H., Park C.B. (2019). Solubility and diffusivity of CO_2_ and N_2_ in TPU and their effects on cell nucleation in batch foaming. J. Supercrit. Fluids.

[50] Ma P., Cai X., Zhang Y., Wang S., Dong W., Chen M., Lemstra P. (2014). In-situ compatibilization of poly(lactic acid) and poly(butylene adipate-co-terephthalate) blends by using dicumyl peroxide as a free-radical initiator. Polym. Degrad. Stab..

[51] Yang J., Chen Z., Xu D., Liu P., Li L. (2021). Enhanced Interfacial Adhesion of Polystyrene Bead Foams by Microwave Sintering for Microplastics Reduction. Ind. Eng. Chem. Res..

