# Supplementary material for: High-Strength Bio-Degradable Polymer Foams with Stable High Volume-Expansion Ratio Using Chain Extension and Green Supercritical Mixed-Gas Foaming
Source: Polymers (Basel). 2023 Feb 10;15(4):895. doi: 10.3390/polym15040895 (PMC9963428; doi:10.3390/polym15040895)
Supplement: Supplementary file 1 [file polymers-15-00895-s001.zip › Supplementary Materials.pdf]

SI Experiment

1.1 Preparation of diverse samples

Blend samples were prepared according to the formula in Table 1 of the main text. Firstly, The PBAT and PLA resins were vacuum-dried for 12 h at 70 ℃ before use. In a Co-rotating twin-screw extruder (SJSP-20X25, Harbin Harp Electrical Technique Co.), PBAT, PLA was blended with a multifunctional epoxy(CE) ADR 4368 of 0.6 wt%, 0.9 wt%, 1.2 wt%, and 1.5 wt%. From the feeding area to the mold section, a total of 5 zones, the temperature of the 5 zones are set to 150 ℃, 160 ℃, 165 ℃, 175 ℃, and 165 ℃ respectively, and the nose temperature is 160 ℃, to obtain stable melt flow, the screw speed is always kept at 60 rpm. The mixed cylindrical lines were obtained, and the cylindrical particle was obtained after cutting.

The samples were hot-pressed at 190 ℃ for 10min and cold-pressed at the cold press for 5min to obtain plates with a thickness of 1mm. These compressed plates were used to test rheological properties, DSC, and TGA.

1.2 Material characterization analysis

1.2.1 Differential scanning calorimetry（DSC）

The thermal performance of diverse samples was performed by DSC was used (TA Instruments, Q20, USA) under a nitrogen flow of 50 mL/min. The diverse samples were rapidly equilibrated to 190°C at 20°C/min, held for 10 min to eliminate

the thermal history, then cooled to -50℃ at 10℃/min, and then reheated to 190℃ at 10℃/min.

1.2.2 Thermogravimetric analysis（TGA）

The degradation temperatures of diverse samples under 50 mL/min nitrogen flow were determined using a thermogravimetric analyzer (TA Instruments, 2950, USA). Sample analysis was performed at a rate of 10℃/min up to 800℃.

1.2.3 Avrami Isothermal crystallization kinetics

The crystallization kinetic of diverse samples was tested by DSC (TA Instruments, Q20, USA) under a 50 mL/min nitrogen flow rate. The crystallization kinetic of samples was analyzed using the Avrami equation. The diverse samples were rapidly equilibrated to 200 ℃ at 10℃/min, held for 10 min to eliminate the thermal history, and then cooled to an isothermal temperature of 90 ℃ at 10 ℃/min and reacted to complete crystallization.

1.2.4 Rheological properties

A rotational rheometer (HAAKE MARS40, Germany) was employed to investigate the dynamic rheological performances of various samples with a parallel plate (25 mm in diameter and a gap of 1 mm) at 190 ℃, the angular frequency (ω) is 0.1 rad/s-100 rad/s, and the strain amplitude is set to 0.5 %. The rheological properties, such as the viscoelasticity of diverse samples, were obtained.

1.2.5 Scanning electron microscopy (SEM)

All samples were immersed in liquid nitrogen for more than 2 h, quickly fractured, and sputtered with gold before observation. Different surface morphologies of non-foamed and foamed samples were obtained using scanning electron microscopy (SEM) (Regulus 8100, JP) at an operating voltage of 5 kV.

1.2.6 Compounding gas adsorption

Selection of PPCE-1.2 for gas adsorption and foaming research. The gravimetric method determined the foaming adsorption behaviors at different compound gas ratios. Firstly, the samples were dried for 12h in an oven at 70℃ and then put into a high-pressure mold with different gas fractions (Pure CO_2, CO_2:N_2=75:25, CO_2:N_2=50:50, CO_2:N_2=25:75, Pure N_2) were introduced into the high-pressure mold separately, the gas ratio above is the pressure ratio. The saturation condition was 16 MPa, 120℃, 30 min. After the quick release of pressure, the samples were quickly put into the analytical balance within 1 min, and the data was recorded.

1.2.7 Mechanical properties of plate testing

Tested according to ISO-844:2014 standard to test samples for the compression strength of samples at 10% and 50% compression. Tested according to ISO 1209-2:2004 standard to test samples for bending strength and fracture load by bending. Tested according to ISO 1856-2000 standard to test samples for permanent deformation in compression. Tested according to ISO 1798:2008 standard to test tensile strength and elongation at break. Five samples were selected for each test, and the average value was taken.

Table

Table S1. PBAT and PLA detailed property tables and test standard.

	Properties (PBAT)	Properties (PLA)	Test standard
Density (g/cm3)	1.24	1.24	ISO 1183
MFI (g/10min，190℃/2.16kg)	≤5	1-4	ISO 1133
Melt Point (℃)	95-135	155-170	ISO3146
Tensile Strength at Break (MPa)	21±2	40-60	ISO 527
Elongation at Break (%)	≥550	3-13	ISO 527
Notched Izod Impact (KJ/m^2)	≥20	4-14	ISO 179

Table S2. Thermal performance parameter of PBAT, PLA, and diverse PPCE samples.

Specimen	T_{c1}[a]	T_{c2}[b]	T_{m1}[c]	T_{m2}[d]	T_{g1}[e]	T_{g2}[f]	X_{c1}[g]	X_{c2}[h]
PBAT	62.09	-	121.47	-	-28.94	-	11.17%	-
PLA	-	111.06	-	156.13	-	60.66	-	38.26%
PPCE-0	79.40	112.18	122.10	158.35	-28.93	61.89	5.49%	27.71%
PPCE-0.6	79.40	112.09	121.93	158.35	-28.76	61.52	5.05%	27.00%
PPCE-0.9	78.99	112.14	121.32	160.75	-28.61	62.12	4.93%	21.99%
PPCE-1.2	78.78	112.81	120.90	161.83	-28.53	62.64	4.66%	22.60%
PPCE-1.5	78.18	113.13	120.23	162.58	-27.98	62.89	3.22%	16.86%

[a] Crystallization temperature of PBAT

[b] Crystallization temperature of PLA

[c] Melting temperature of PBAT

[d] Melting temperature of PLA

[e] Glass transition temperature of PBAT

[f] Glass transition temperature of PLA

[g] Crystallinity of PBAT

[h] Crystallinity of PLA

Table S3. Thermal decomposition properties of diverse samples.

Sample	Thermal decomposition temperatures, °C					Residual weight (at 800°C)
	$T_{5\%}$[a]	$T_{10\%}$[b]	$T_{50\%}$[c]	T_{max1}[d]	T_{max2}[e]	
PBAT	364.17	374.17	398.67	-	399.17	3.70%
PLA	302.08	315.67	343.21	348.52	-	3.01%
PPCE-0	340.67	350.17	395.5	329.17	398.83	2.77%
PPCE-0.6	334.5	344.83	389.83	353.17	398.17	1.67%
PPCE-0.9	338.5	347.33	392.50	353.17	398.50	0.91%
PPCE-1.2	334.83	345.50	389.83	353.67	398.00	1.85%
PPCE-1.5	336.67	346.17	391.17	353.83	398.33	3.48%

[a] 5% thermal weight loss temperature

[b] 10% thermal weight loss temperature

[c] 50% thermal weight loss temperature

[d] Exothermic peak (PLA)

[e] Exothermic peak (PBAT)

Table S4. Orthogonal experiment design of PPCE-1.2.

Experiment	Temp(A)	Pressure(B)	Time (C)	Blank(D)	VER
1	A1	B1	C1	D1	5.54
2	A1	B2	C2	D2	8.92
3	A1	B3	C3	D3	13.33
4	A2	B1	C2	D3	10.33
5	A2	B2	C3	D1	13.93
6	A2	B3	C1	D2	12.28
7	A3	B1	C3	D2	20.67
8	A3	B2	C1	D3	22.14
9	A3	B3	C2	D1	10.08
K1	27.79	36.54	39.96	29.55	
K2	36.54	44.99	29.33	41.87	
K3	52.89	35.69	47.93	45.80	
k1	9.26	12.18	13.32	9.85	
k2	12.18	15.00	9.78	13.96	
k3	17.63	11.90	15.98	15.27	
R	8.37	3.10	6.20	5.42	
Best quality level	A3	B2	C3		
Optimal combination	120℃，14MPa，30min				

Temperature A1−A3: 110, 115, and 120 °C; Pressure B1−B3: 12, 14, and 16 MPa; C1−C3: 10, 20 and 30min; D1−D3: Blank control. K: average value of each factor at different levels. R: extremum of each factor.

Table S5. Results of foaming parallel experiments in a specific interval of foaming conditions.

Temperature/(℃)	Pressure/(MPa)	Time/(min)	VER
110	12	10	5.54
110	12	20	8.16
110	12	30	8.55
110	14	10	5.82
110	14	20	8.92
110	14	30	9.25
110	16	10	6.14
110	16	20	12.92
110	16	30	13.33
115	12	10	7.9
115	12	20	10.33
115	12	30	10.51
115	14	10	11.07
115	14	20	13.05
115	14	30	13.93
115	16	10	12.28
115	16	20	17.22
115	16	30	17.71
120	12	10	17.46
120	12	20	20.33
120	12	30	20.67
120	14	10	22.14
120	14	20	22.55
120	14	30	22.96
120	16	10	9.92
120	16	20	10.08
120	16	30	9.84

Table S6. CO_2 and N_2 gas adsorption amounts.

Gas component	Solubility (total blowing agent/100g polymer) [g]
100%CO_2	5.36
75%CO_2+25%N_2	4.95
50%CO_2+50%N_2	3.45
25%CO_2+75%N_2	1.98
100%N_2	0.53

Figure

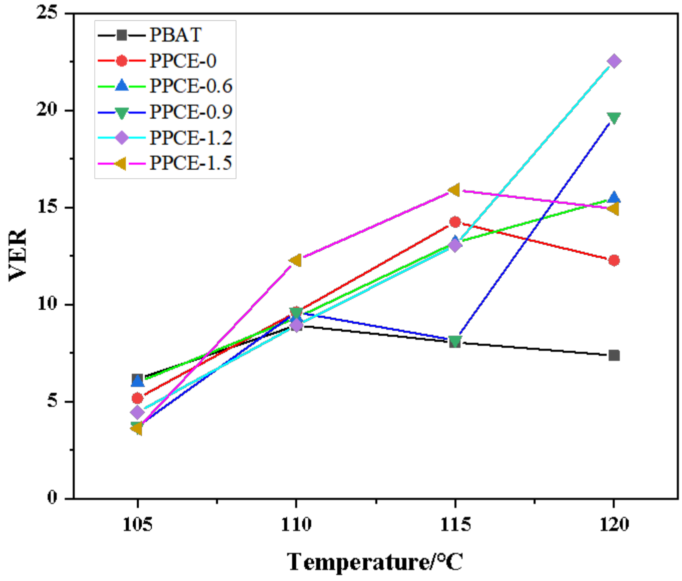

Figure S1. Diverse samples saturated with 14 MPa CO_2 saturated atmosphere for 30min and foamed at different foaming temperatures.

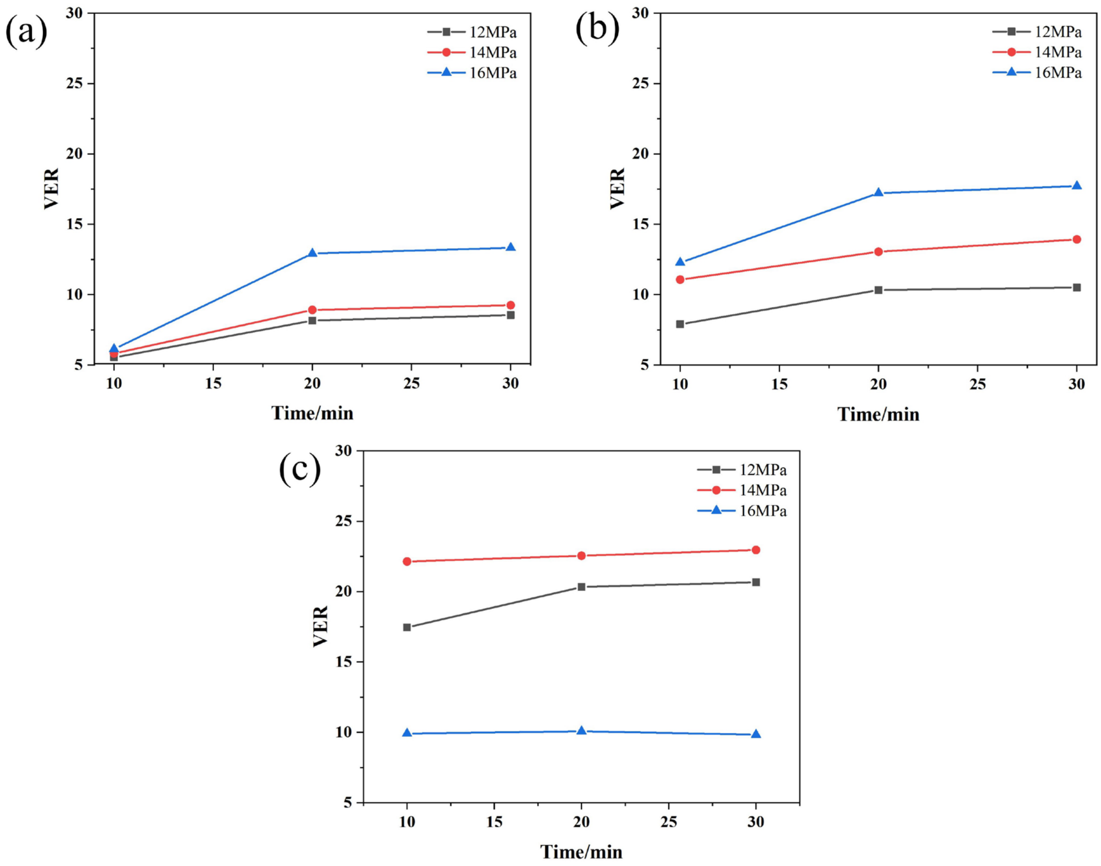

Figure S2. Parallel experiments on PPCE-1.2: (**a**) 110 °C, (**b**) 115 °C, (**c**) 120 °C.

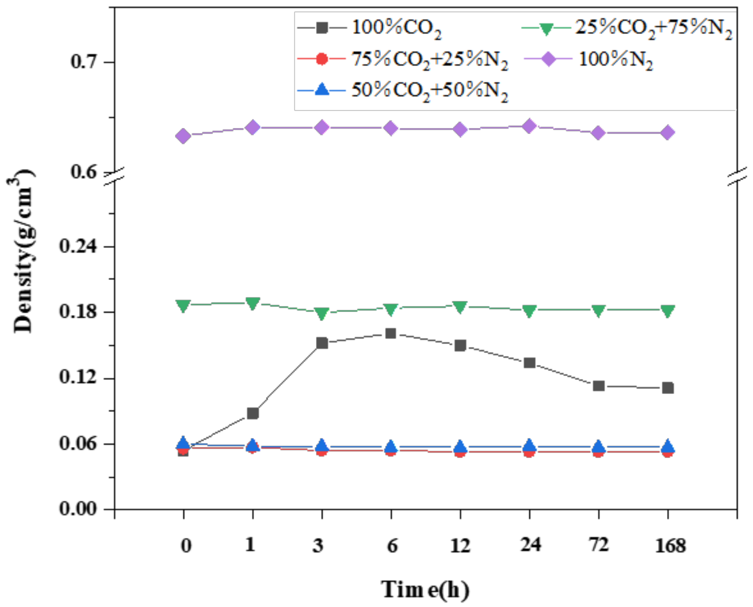

Figure S3. Density variation of foam maturation process under different gas conditions.
